# Contextualizing guidelines for the health system of Cyprus: Experiences and lessons learnt

**DOI:** 10.1002/gin2.70013

**Published:** 2025-02-05

**Authors:** Panayiotis Kouis, Hugh McGuire, Monika Kyriacou, Anneza Yiallourou, Anastasis Sioftanos, Ourania Kolokotroni, Haris Achilleos, Craig Grime, Pilar Pinilla‐Dominguez, Giorgos Giallouros, Christina Englezou, Contextualization group, George Athanasiou, Elias Avraam, Mary Avraamidou, Anastasios Bagourdis, Charis Charilaou, Stelios Christodoulou, Natasa Christofides, Theodoros Christophides, Vasilis Constantinidis, Dimitris Dimitriou, Stella Eleftheriadou, Christina Flourou, Savvas Frangos, Kyriakos Frantzeskou, Costas Hoplaros, Kyriakos Ioannou, Nikolaos Kadoglou, Chara Kalogirou, Vasileios Karathanos, Andreas Kostis, Andreas Kousios, Ekaterini Lambrinou, Emily Mavrokordatou, Michael Michael, Nicos Mitsides, Chrystalleni Mylonas, Georgia Orphanou, Elias Papasavvas, Doros Polydorou, Stavros Stavrou, Elena Theofanous, Despo Theodorou, Giannis Thrasyvoulou, Chryssa Tziakouri‐Shakalli, Theodora Vassiliou, Alexis Vrachimis, Panayiotis K. Yiallouros, Georgios K. Nikolopoulos

**Affiliations:** ^1^ Medical School University of Cyprus Nicosia Cyprus; ^2^ National Institute for Health and Care Excellence London UK; ^3^ Health Insurance Organization Nicosia Cyprus; ^4^ School of Health Sciences Cyprus University of Technology Limassol Cyprus; ^5^ Paediatric Accident & Emergency Department, Nicosia General Hospital State Health Services Organization Nicosia Cyprus; ^6^ Department of Quantitative Methods for Economics and Management University of Las Palmas de Gran Canaria Las Palmas Spain

**Keywords:** adaptation, contextualization, Cyprus, guidelines, healthcare system, health system transition, NICE guidelines

## Abstract

**Background:**

Cyprus is undergoing a major health reform with the recent establishment of the General Healthcare System (GHS). The GHS offers equal healthcare access through one primary insurer (Health Insurance Organization [HIO]) and benefits from a wide collaborative network of public and private healthcare providers. However, unwanted variation in practice makes this transition challenging. Healthcare guidelines could decrease these variations in practice, but Cyprus lacks the capacity to develop them de novo. Through a collaboration with the National Institute for Health and Care Excellence (NICE) in the United Kingdom, the contextualization of NICE guidelines and the derivation of local quality indicators are carried out. This study presents the methodology and experience of contextualizing the first three NICE guidelines and deriving associated quality indicators in Cyprus.

**Methods:**

HIO managed the guideline contextualization with the support of a Guidelines Secretariat. For each guideline, a local topic expert committee (TEC) was recruited. Through a series of meetings, followed by public consultation, each TEC made contextual changes to the guideline and derived relevant quality indicators. During this process, NICE assured quality by overseeing several elements of the contextualization procedure such as TEC membership, proposed changes and justification and derived quality indicators.

**Results:**

Between 2022 and 2024, three NICE guidelines, NG196 on Atrial fibrillation (AF), NG230 on Thyroid cancer (TC) and NG203 on Chronic kidney disease (CKD), were contextualized through the modification of several individual guideline recommendations (21/79 [26.6%] in AF, 37/67 [55.2%] in TC and 62/217 [28.6%] in CKD). In parallel, NICE quality indicators were screened for applicability and feasibility in Cyprus while additional indicators were developed if required.

**Conclusion:**

In a country with limited experience in guideline development, a supervised and systematic process supported by an established organization ensures quality, is less resource intensive and builds capability for the sustainability of the process.

## Highlights

- Cyprus faces unwanted variation in clinical practice and currently lacks the capacity to develop healthcare guidelines de novo.
- Through a supervised adaptation process, whose quality was assured by the National Institute for Health and Care Excellence (NICE) in the United Kingdom, the Cyprus Health Insurance Organization involved key local healthcare and societal stakeholders and successfully contextualized three NICE guidelines and 17 quality indicators for Cyprus.
- The contextualization of NICE guidelines was a timely and efficient process. It provided training opportunities for those involved and demonstrated the benefits of capacity and capability building in countries with limited experience in developing, adapting and implementing evidence‐based guidelines.

## 1 INTRODUCTION

It is widely accepted that medical practice should be evidence‐based, guided by experts' knowledge and experience as well as by scientific evidence.^1^ Evaluating scientific evidence can be complex for individual practitioners nowadays, due to the significant rise in medical and public health studies published over the last decades. This has created the need for systematic identification, collation and careful appraisal of the accrued scientific information.^2^ Guidelines and specific practice‐oriented recommendations, based on systematic reviews, help address this problem. Many groups and networks have been operating at national and international levels for formulation of guidelines,^3^ which have increasingly become an integral part of clinical practice. Guidelines also influence the purchase and funding of healthcare services and can inform quality standards and indicators that can be used to assess healthcare provision.^4^ Eventually, guidelines can help educate health professionals and support patients to make informed decisions on their health.^5^, ^6^

Nevertheless, developing new guidelines is time‐consuming and resource‐intensive.^7^, ^8^ In contrast, the adaptation of existing guidelines for local use is a faster, resource‐efficient approach that reduces the duplication of effort and can facilitate uptake by giving ownership to those engaged in the development process.^9^ Even in cases where the evidence base is identical, cultural and organizational differences across and within countries can result in sensible variations in recommendations. Consequently, guidelines developed in one context may not be directly applicable to another without thorough contextual adaptation.^10^ During guideline adaptation, consideration is given to local factors, such as setting; specific needs, priorities, legislation, policies and resources; and scopes of practice within the local health services to fit into the local setting.^11^ Different systematic approaches to address these critical concepts in guideline adaptation have been developed and they include the ADAPTE Collaboration process^10^ and resource toolkit as well as the GRADE‐ADOLOPMENT methodology within the GRADE framework.^12^

Cyprus is a country in the eastern Mediterranean basin and a member state of the European Union. Following long‐term discussions, a major health reform materialized in the country with the establishment of the General Healthcare System (GHS) in 2019.^13^ GHS offers equal access to healthcare services for all citizens through one primary insurer (Health Insurance Organization [HIO]). It has a wide collaborative network of primary, secondary and tertiary healthcare providers, both from the public and the private sector. Transition to GHS is expected to be sustainable, provide high‐quality services and improve long‐term survival, healthy life years and quality of life outcomes. While the first evaluations of the new system are positive, some challenges, including unwanted variation in medical practice, have been identified. Training of medical practitioners in Cyprus is not based on a uniform educational system that aims at efficiency, as a prerequisite for long‐term sustainability of the system. Therefore, the use of guidelines, adapted to the local context, should help decrease disparities in healthcare services, optimize the efficient use of resources and allow for effective clinical audits across the country.

While guidelines are needed in Cyprus, the country lacks local capacity to develop them. Within this context, HIO, the University of Cyprus (UCY) and the National Institute for Health and Care Excellence (*NICE* in the United Kingdom [UK]—https://www.nice.org.uk/) entered a collaboration to address these issues. NICE has been developing guidelines, through a rigorous mechanism, over the last 25 years. In addition, NICE supports the contextualization of its guidelines to other jurisdictions through a systematic and supervised process that is based on the ADAPTE framework with modifications.^10^ This contextualization process also enables capability building in guideline and associated quality indicator development processes and methods while it also facilitates local ownership of the contextualized guidelines.

The aim of this work is to present the methodology and experience of contextualizing three NICE guidelines and deriving associated quality indicators in Cyprus.^14^

## 2 OVERVIEW OF NICE GUIDELINE CONTEXTUALIZATION PROCESS

The NICE guideline contextualization process involves several teams:

- The host organization (HIO and Guidelines Secretariat at UCY) which manages the guideline process including committee recruitment and management, stakeholder consultation and guideline publication and dissemination.
- The locally recruited topic/technical expert committee (TEC) which contextualizes the guideline and derives quality indicators.
- NICE which provides quality assurance and support.

The Guideline Secretariat at UCY consists of methodologists and clinicians with a broad knowledge of the Cyprus healthcare system and offers administrative and methodological support to the TEC activities. The TEC consists of clinical experts and patient representatives for each specific guideline topic. NICE provides quality assurance and capability building as well as support around stakeholder engagement and public consultation processes.

In general, the guideline topics prioritized by HIO and Secretariat, following feedback received from local medical scientific societies and local health indicators, are considered against the NICE portfolio as follows:

- Is the equivalent NICE guideline up to date?
- Is the NICE guideline being updated at present or in the near future?

Once the candidate guidelines have been identified, the HIO recruits the TEC, which makes contextual changes to the NICE scope and the NICE guideline and derives relevant quality indicators. The contextualization process includes a series of on‐site TEC meetings, followed by a public consultation process, so stakeholders can provide input into the contextualized guideline and proposed indicators. The TEC meetings include an induction to the contextualization process (preparatory meeting), a point‐by‐point discussion of modifications to the scope and guideline, a discussion of the priorities and testing requirements for quality indicators and, at a final postconsultation meeting, a review of consultation comments and finalizing the contextualized guideline. During this process, NICE provides quality assurance by reviewing several elements of the contextualization procedure such as TEC membership, proposed modifications and justifications and derived quality indicators. A full description of the elements that NICE reviews is provided in Supporting Information S1: Table S1.

## 3 IMPLEMENTATION OF THE NICE GUIDELINE CONTEXTUALIZATION PROCESS IN CYPRUS

### 3.1 Preparation for guideline contextualization

In April 2021, the collaboration with NICE began and HIO invited its health system stakeholders, such as the Cyprus Medical Association, to join this activity. Following a scoping exercise to understand the healthcare landscape in Cyprus, several activities to support the institutionalization of guidelines in Cyprus were agreed. These included capability‐building seminars for guideline development and associated support mechanisms with topics such as managing a guideline program, ensuring transparency in decision‐making, committee recruitment and management and managing declarations of interest. Many of these sessions were delivered as part of a 2‐day stakeholder engagement and awareness‐raising event titled ‘Implementing evidence‐based guidance and quality standards and indicators in GHS’ that was held in Cyprus in December 2021.

Following this event and through negotiation with key stakeholders including medical societies and patient organizations, HIO established a process to recruit TEC members that included the nomination of potential TEC members by medical societies and patient organizations, and the final selection by HIO. There was an effort to include both the public and private healthcare sectors and ensure that all relevant medical societies and patient organizations would be actively involved in the TEC activities.

The NICE portfolio of guidelines was then assessed. Several topics were prioritized by the HIO and Secretariat based on criteria including health burden, expected variations in practice and feasibility of implementation. Following consideration against the NICE portfolio, the following three guidelines were selected for contextualization between 2022 and 2024:

- NICE guideline [NG196] atrial fibrillation (AF): diagnosis and management.^15^
- NICE guideline [NG203] chronic kidney disease (CKD): assessment and management.^16^
- NICE guideline [NG230] thyroid cancer (TC): assessment and management.^17^

### 3.2 Participation/meetings

**Figure 1:**
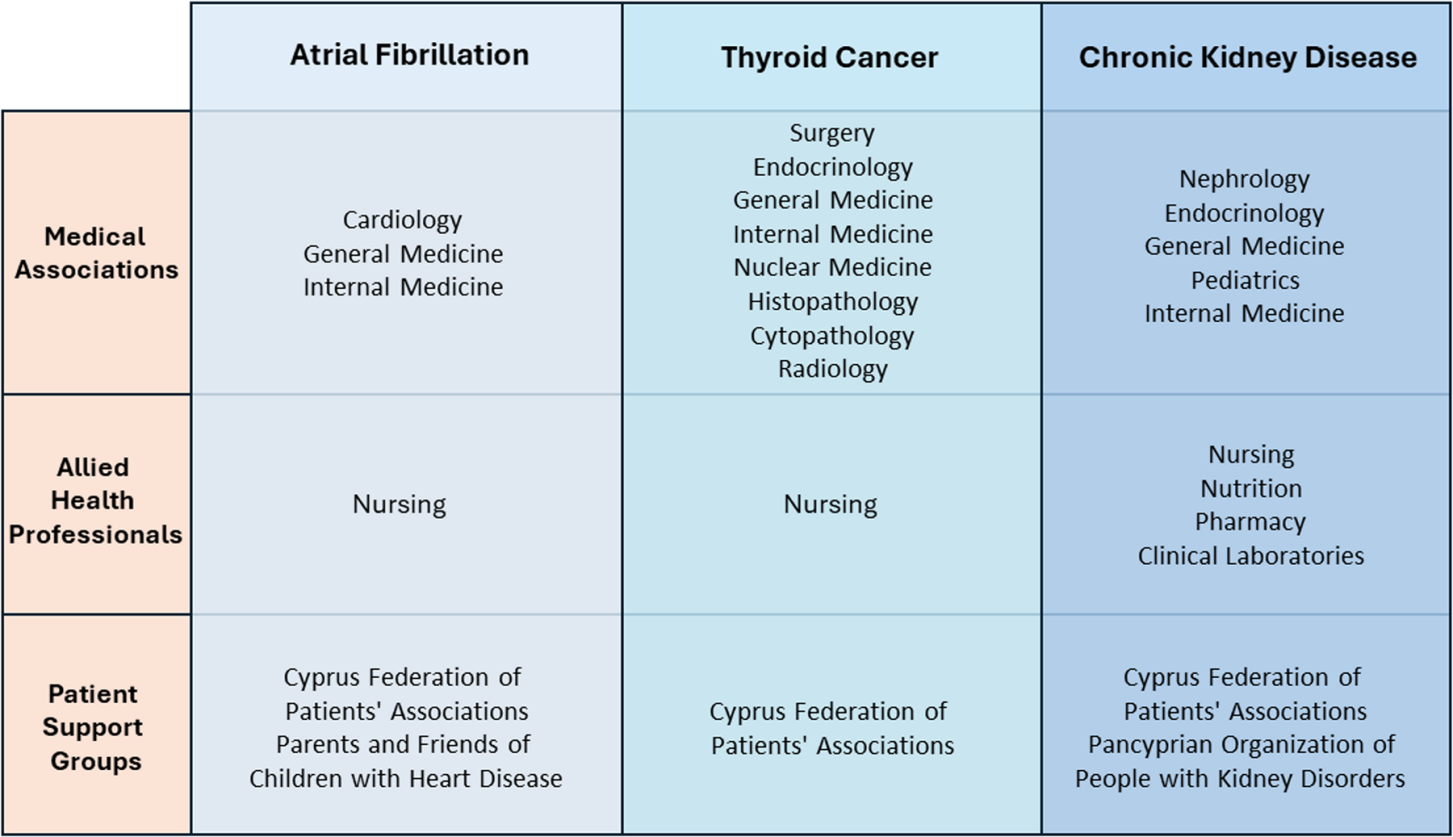
AF, TC and CKD technical expert committees' composition: member composition of the atrial fibrillation, thyroid cancer and chronic kidney disease technical expert committees. AF, atrial fibrillation; CKD, chronic kidney disease; TC, thyroid cancer.

In total, 20 stakeholder organizations (38 individual representatives) were involved in the contextualization process for the first three guidelines (Figure 1). Most of the stakeholders participated in the contextualization of one guideline, according to their expertise, although there were some organizations (Cyprus Federation of Patients' Associations, Cyprus Association of Family Doctors or Cyprus Society of General Medicine) which participated in all three guidelines. For two out of three guidelines, a total of six TEC meetings were carried out as originally scheduled, while the contextualization of the thyroid cancer guideline required an additional meeting (during the point‐by‐point contextualization phase). The full contextualization process lasted between 9 months (AF guideline) and 11 months (TC and CKD guidelines) and overall attendance in TEC meetings among members was high (mean attendance: 75.3%). TEC members unable to attend some meetings in person were kept informed through electronic communication of materials and meetings' minutes.

### 3.3 Modification of guidelines

Following the first preparatory meeting, each TEC member was asked to review the guideline scope and materials, and in the subsequent meetings, a detailed point‐by‐point discussion of the guideline ensued. Overall, these discussions led to modifications in the introductory section of the three guidelines and modification of several individual recommendations (21/79 [26.6%] in AF, 37/67 [55.2%] in TC and 62/217 [28.6%] in CKD]. The modified recommendations, accompanying justifications and supporting evidence provided by the TEC, were reviewed by NICE (both by NICE methodology and clinical experts) and the percentage of modified recommendations for each contextualized guideline corresponds to the number of modified recommendations, retained in the final contextualized version following NICE review, out of the total recommendations included in the guideline. Furthermore, the TEC updated the supporting rationale and impact section and added new Appendices in each contextualized guideline (3 in AF, 2 in TC and 1 in CKD).

The TEC also added information on the guideline format and the use of specific wording to denote the strength of each recommendation (e.g., ‘must’ or ‘must not’ for mandatory recommendations, ‘offer’ or ‘do not offer’ for strong recommendations and ‘consider’ for weak recommendations). In addition, all hyperlinks in the contextualized document were replaced with relevant text either within the body of the guideline or using relevant Appendices. This allows the contextualized guideline to be read as a stand‐alone document.

### 3.4 Types of modifications

The main effort of contextualization focused on modifying specific recommendations, the removal of recommendations or elements of recommendations that were not applicable to the local setting such as those related to medications, pathways or services not marketed or offered in Cyprus, the conversion of recommended thresholds and doses to units used by laboratories and practitioners in Cyprus and the replacement of hyperlinked UK resources for drug safety with corresponding resources from Cyprus and the European Union.

However, some modifications required a more detailed discussion and additional justification that relied on high‐quality published studies (other international guidelines, systematic reviews, randomized control trials), as well as the current clinical practice and cost estimates in Cyprus (Table 1). This approach also applied to recommendations that were underpinned by cost‐effectiveness evaluations relevant to the UK National Health System (NHS) context. In these cases, each TEC considered available data on resource use and cost estimates in the Cyprus healthcare system when justifying its edits. Characteristic examples of the different contextual modifications are described in Table 1.

**Table 1:** Examples of contextualized recommendations and associated justification.

#	CG	Recommendation (# CG)	Original wording	Contextualized wording	Justification
1	Atrial fibrillation (AF)	1.3.1 (1.3.1)	Perform transthoracic echocardiography (TTE) in people with atrial fibrillation:	Perform transthoracic echocardiography (TTE) in people with atrial fibrillation:	The AF TEC was in favour of all newly diagnosed AF patients undergoing echocardiography. This position is in line with previous expert consensus statements and guidelines. https://pubmed.ncbi.nlm.nih.gov/26864186/ https://www.jacc.org/doi/full/10.1016/j.jacc.2006.07.009 In addition, this approach reflects the current practice in Cyprus and as a result, it is not expected that the number of patients that will be offered TTE will change.
			For whom a baseline echocardiogram is important for long‐term management	When first diagnosed with atrial fibrillation	
2	AF	1.7.19 (1.7.18)	If drug treatment is unsuccessful, unsuitable or not tolerated in people with symptomatic paroxysmal or persistent atrial fibrillation:	If drug treatment is unsuccessful, unsuitable or not tolerated in people with symptomatic paroxysmal or persistent atrial fibrillation:	The AF TEC expressed the opinion that the different approaches of ablation (point by point and cryoballoon/laser balloon) were found to be of equal effectiveness according to recent evidence; for this reason, they should be treated equally. https://pubmed.ncbi.nlm.nih.gov/29564527/ https://pubmed.ncbi.nlm.nih.gov/36184349/ https://pubmed.ncbi.nlm.nih.gov/31630538/ The AF TEC acknowledged that the order of the procedures in the original NICE guideline resulted from different costs within the NHS system which resulted in different cost‐effectiveness estimates between the different ablation approaches. However, in Cyprus, the cost differences between the procedures are negligible as confirmed by HIO and allow for equal recommendations for the three procedures.
			Consider radiofrequency point‐by‐point ablation or If radiofrequency point‐by‐point ablation is assessed as being unsuitable, consider cryoballoon ablation or laser balloon ablation	Consider radiofrequency point‐by‐point ablation or cryoballoon ablation or laser balloon ablation	
3	Thyroid cancer (TC)	No recommendation (1.2.15)	None	Consider deferring FNA until after pregnancy, taking into account:	The TC TEC introduced this additional recommendation to make a special reference to FNA during pregnancy. Special reference to FNA during pregnancy was considered important since differentiated thyroid cancer has a very good prognosis and special attention needs to be given to outweigh the benefit of diagnosing this type of cancer during pregnancy versus the psychological burden imposed on the patient and family to be informed about a thyroid cancer diagnosis and having to make decisions regarding the pregnancy itself. This modification was based on the local experience in patient management, existing cultural issues and attitudes of the Cyprus population. The TEC argued that local attitudes often do not allow the prudent management of a patient with a positive FNA result (active surveillance and deferring surgery) with several women choosing to terminate pregnancy or operate during pregnancy. Although relevant published data are not available in Cyprus now to further demonstrate this point, several TEC members highlighted that they are aware of similar cases in the past with pregnant women terminating pregnancies or being operated during pregnancy. The TEC also argued that the wording of this modification highlights a joint decision process between the woman and the involved medical specialists, a process which is highly applicable in the local context, where small distances and small number of specialists allow for efficient collaboration and close and careful management of the pregnant patient.
				The risk of delaying FNA The risk to the pregnancy The rate of disease progression	
				The obstetrician, surgeon and endocrinologist should discuss these factors and a joint decision should be reached in discussion with the pregnant woman.	
4	TC	1.4.3 (1.4.3)	Use *dynamic risk stratification* to determine further management at 9 to 12 months after completion of initial *RAI ablation*, as follows:	Use *dynamic risk stratification* to determine further management at 9 to 12 months after completion of initial *RAI ablation*, as follows:	The TEC has added the definitions for excellent and intermediate response to TSH suppression using as a source the NICE evidence documentation from NG230 Thyroid cancer: assessment and management: Evidence review M 19/12/2022. In addition, the TEC added the corresponding references to the published literature.
			Reduce TSH suppression to achieve a TSH level of between 0.3 mIU/litre and 2.0 mIU/litre and continue this for life in people with an excellent response to treatment. Continue TSH suppression to achieve a TSH level of between 0.1 mIU/litre and 0.5 mIU/litre in people who have an intermediate response to initial treatment.	Reduce TSH suppression to achieve a TSH level of between 0.3 mIU/litre and 2.0 mIU/litre and continue this for life in people with an excellent response to treatment. Excellence response is defined as no clinical, biochemical or structural evidence of disease: negative imaging and either suppressed Tg, 0.2 ng/mL or stimulated Tg, 1 ng/mL.^18^ Continue TSH suppression to achieve a TSH level of between 0.1 mIU/litre and 0.5 mIU/litre in people who have an intermediate response to initial treatment. An intermediate response is defined as nonspecific biochemical (i.e., suppressed Tg 0.2–1 ng/mL or stimulated Tg 1–10 ng/mL or stable/declining anti‐Tg antibody levels) or structural findings that cannot be confidently classified as either benign or malignant.^18^	
5	Chronic kidney disease (CKD)	1.6.4 (1.6.4–1.6.5)	1.6.4. Follow the recommendations on treating hypertension in *NICE's guideline on hypertension in adults* for adults with CKD, hypertension and an ACR of 30 mg/mmol or less (ACR categories A1 and A2).	1.6.4. For patients who are extremely elderly (>90 years) or have multiple comorbidities, a number of additional factors should be considered to set the target levels for blood pressure control.	The CKD TEC introduced a new recommendation 1.6.4 to highlight that when setting target levels for blood pressure control in extremely elderly individuals, other comorbidities should be considered. This addition highlights the importance of carefully reviewing the patient's profile including other comorbidities and corresponding concurrent medications, before setting the target levels for blood pressure control. Although there is lack of published evidence, the TEC members agreed that the prevalence polypharmacy is expected to be high among extremely elderly individuals and people with multiple comorbidities in Cyprus. For this reason, the intensity of blood pressure control and the prescription of antihypertensive medication should be considered in light of this information to reduce the possibility of adverse drug interactions and improve medication adherence.
				1.6.5. Offer lifestyle interventions and consider starting antihypertensive drug treatment in adults with CKD, hypertension and an ACR of 300 mg/gr or less (ACR categories A1 and A2).	
					For contextualized point 1.6.5, the TEC added the statement ‘Offer lifestyle interventions and consider starting antihypertensive drug treatment’ to reflect the nature of recommendations 1.4.1–1.4.14 of NICE guideline NG136. The link to NICE guideline NG136 was removed so that the contextualized guideline can be read as a stand‐alone document. TEC also converted ACR values and units to the format used in Cyprus (from mg/mmol to mg/gr). The conversions were carried out using an established *online calculator* and rounded to conventional numbers used in the clinical setting in Cyprus.
6	CKD	16.9 (1.6.11)	For adults with CKD but without diabetes:	For adults with CKD but without diabetes:	The TEC modified this recommendation to suggest the use of SGLT2 inhibitors added in patients with CKD and no diabetes in line with the two recent NICE Technology Appraisals (TAGs, one on dapagliflozin—Technology appraisal guidance [TA775] and one on empagliflozin—technology appraisal guidance [TA942]). https://www.nice.org.uk/guidance/ta775/chapter/1-Recommendations https://www.nice.org.uk/guidance/ta942 The TEC also acknowledged that the edits in this recommendation in the contextualized guideline for Cyprus were primarily based on the clinical effectiveness of these drugs as specific information on their cost‐effectiveness in the Cyprus setting was not available and was not possible to be collected as part of the contextualization process. Nevertheless, the TEC members as well as HIO expect that the cost will be within an acceptable margin and will not outweigh its potential clinical benefits. In addition, as part of the discussion, it was clarified by HIO representatives that HIO follows a flexible drug reimbursement approach that allows, in cases of more expensive medication, to offer the medication with a co‐payment by the patient. As such, from a healthcare system perspective, the cost‐effectiveness of the medication can be maintained.
			Refer for nephrology assessment and offer an ARB or an ACE inhibitor (titrated to the highest licensed dose that they can tolerate), if ACR is 70 mg/mmol or more Monitor in line with recommendations 1.3.1 and 1.3.4 if ACR is above 30 but below 70 mg/mmol; consider discussing with a nephrologist if eGFR declines or ACR increases.	Refer for nephrology assessment and offer an ARB or an ACE inhibitor (titrated to the highest licensed dose that they can tolerate), if ACR is 500 mg/gr or more. Offer SGLT‐2 inhibitors for treating chronic kidney disease (CKD) in adults. It is recommended only if: It is an add‐on to optimized standard care including the highest tolerated licensed dose of angiotensin‐converting enzyme (ACE) inhibitors or angiotensin‐receptor blockers (ARBs), unless these are contraindicated. ∘ People have an estimated glomerular filtration rate (eGFR) of 20 mL/min/1.73 m^2^ at the start of treatment and: ∘ Have a urine albumin‐to‐creatinine ratio (ACR) of 200 mg/gr or more. Monitor in line with recommendations 1.3.1 and 1.3.4 if ACR is above 300 but below 500 mg/gr; consider discussing with a nephrologist if eGFR declines or ACR increases.	

Abbreviations: ACE angiotensin‐converting enzyme; ACR, albumin creatinine ratio; AF, atrial fibrillation; ARB, angiotensin receptor blockers; CG, contextualized guideline; CKD, chronic kidney disease; eGFR, estimated glomerular filtration rate; FNA, fine needle aspiration; HIO, Health Insurance Organization; NHS, UK National Health System; NICE, National Institute for Health and Care Excellence in the United Kingdom; RAI, radioactive iodine; SGLT2, sodium‐glucose co‐transporter 2; TEC, topic/technical expert committee; TSH, thyroid stimulating hormone.

### 3.5 Selection and development of indicators

Screening of the NICE library of indicators retrieved a total of 26 potential indicators, relevant to the three contextualized guidelines. A total of nine indicators were applicable to the AF guideline, six to the TC guideline and 11 to the CKD guideline. These 26 potential indicators were initially assessed by TEC and 13 indicators were excluded from further contextualization.

**Figure 2:**
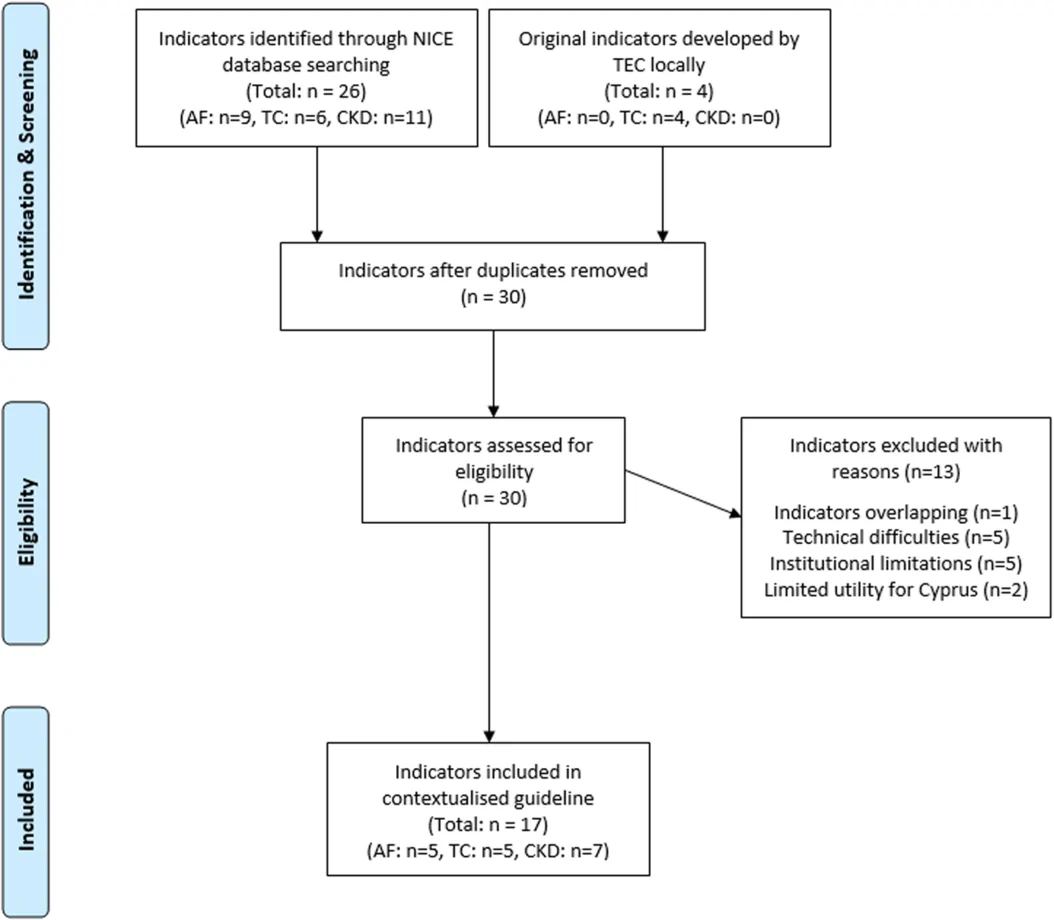
Modified Prisma flow chart for selection of quality indicators: Modified Prisma flow chart demonstrating the selection of quality indicators with reasons for exclusion. AF, atrial fibrillation; CKD, chronic kidney disease; NICE, National Institute for Health and Care Excellence in the United Kingdom; TC, thyroid cancer; TEC, topic/technical expert committee.

There were no TC‐specific NICE indicators and given the absence of systematic appraisal of surgical outcomes by the relevant professional bodies in Cyprus, the TC TEC decided to develop four indicators around surgical management. These additional indicators focused on the assessment and frequency of permanent vocal cord paralysis and permanent hypoparathyroidism following thyroidectomy/thyroid surgery. Following the initial selection and discussion of the indicator format (exclusions, relevant GHS electronic system codes, implementation setting), a set of business rules was developed for each indicator. In total, the final number of indicators for each guideline was 5 for AF, 5 for TC and 7 for CKD (Figure 2). Business rules included data set specification in the form of time periods, patient selection criteria, clinical code clusters and data extraction criteria, as well as the data export logic rule(s) based on a series of Boolean operators.^19^ A sample of indicators was tested before the implementation of the guidelines to assess the feasibility and recognize technical faults. Table 2 summarizes all contextualized indicators and provides examples of the indicator testing output, while Supporting Information S1: Table 2 summarizes all the excluded indicators and provides the reason(s) for exclusion.

**Table 2:** Summary of selected indicators for the atrial fibrillation (AF), thyroid cancer (TC) and chronic kidney disease (CKD) contextualized guidelines (CG).

#	CG	NICE reference	Contextualized title	Exclusions	Indicator testing results
1	AF	CCG55	The proportion of patients admitted to hospital for stroke with a pre‐existing diagnosis of atrial fibrillation, who were on anticoagulation.	No exclusions apply to this indicator.	**Testing (evaluation) date**: 31/12/2022 **Denominator:** 204 patients admitted to hospital with a primary diagnosis of stroke, who had a pre‐existing diagnosis of atrial fibrillation **Numerator:** Out of those in the denominator, 101 had taken at least one of the related drugs within 3 months of admission for stroke. **Calculation:** Numerator/denominator) * 100 **Indicator result:** 49.5%
2	AF	NM146GP	The percentage of patients registered at the practice aged 65 years and over who have been diagnosed with one or more of the following conditions: coronary heart disease, heart failure, hypertension, diabetes, chronic kidney disease, peripheral arterial disease or stroke/transient ischaemic attack who have had a pulse rhythm assessment in the preceding 12 months.	People with diagnosed atrial fibrillation.	Not performed
3	AF	NM147GP	The percentage of patients with atrial fibrillation, currently treated with an anticoagulant, who did not have an assessment in the preceding 12 months of renal function, creatinine clearance, full blood count (FBC) and liver function tests (LFTs) as appropriate for their anticoagulation therapy.	No exclusions apply to this indicator.	Not performed
4	AF	NM81GP	The percentage of patients with atrial fibrillation in whom stroke risk has been assessed using the CHA2DS2‐VASc score risk stratification scoring system in the preceding 12 months (excluding those patients with a previous CHA2DS2‐VASc score of 2 or more).	Patients with atrial fibrillation with a previous CHA2DS2‐VASc score of 2 or more.	Not performed
5	AF	NM82GP	In those patients with atrial fibrillation with a record of a CHA2DS2‐VASc score of 2 or more, the percentage of patients who are currently treated with anticoagulation drug therapy.	No exclusions apply to this indicator.	Not performed
6	TC	NM62	The percentage of patients with thyroid cancer diagnosed within the preceding 15 months who have a review recorded as occurring within 3 months of the practice receiving confirmation of the diagnosis.	Patients who have not responded to at least two cancer care review invitations made at least 7 days apart.	Not performed
7	TC	Additional A	The percentage of patients who have undergone thyroid cancer surgery in the previous 12 to 24 months, who have been diagnosed with permanent postoperative vocal cord paralysis.	Patients with pre‐existing vocal cord paralysis or other vocal cord abnormality.	**Testing (evaluation) date**: 31/12/2023 **Denominator:** Eight patients have undergone thyroid cancer surgery in the previous 12–24 months and have been assessed for vocal cord paralysis 6 months or more after the surgery date. **Numerator:** Out of those in the denominator, 0 have been diagnosed with permanent postoperative vocal cord paralysis. **Calculation:** Numerator/denominator) * 100 **Indicator result:** 0%
8	TC	Additional B	The percentage of patients who have undergone thyroid cancer surgery between 12 and 24 months before indicator assessment and have been diagnosed with permanent postoperative hypoparathyroidism in the preceding 12 months.	No exclusions apply to this indicator.	**Testing (evaluation) date**: 31/12/2023 **Denominator:** 27 patients who have undergone thyroid cancer surgery between 12 and 24 months before the time of indicator assessment and have been assessed for hypoparathyroidism 12 months or more after the surgery date. **Numerator:** Out of those in the denominator, 1 has been diagnosed with permanent postoperative hypoparathyroidism in the preceding 12 months. **Calculation:** Numerator/denominator) * 100 **Indicator result:** 3.7%
9	TC	Additional C	The percentage of patients who have undergone thyroid cancer surgery in the previous 12–24 months, who have been assessed for permanent postoperative vocal cord paralysis, 6 months or more after the surgery date.	No exclusions apply to this indicator.	Not performed
10	TC	Additional D	The percentage of patients who have undergone thyroid cancer surgery between 12 and 24 months before indicator assessment and have been assessed for postoperative hypoparathyroidism 12 months or more after the surgery date.	No exclusions apply to this indicator.	Not performed
11	CKD	NM109	The percentage of CKD‐coded patients, whose notes have a record of referral for urine albumin:creatinine ratio (or protein:creatinine ratio) test in the preceding 12 months.	Patients with CKD category G1 or G2 who do not have any risk factors.^a^	**Testing (evaluation) date**: 31/12/2023 **Denominator:** 19,188 patients are CKD stage 3–5 or CKD stage 1–2 but have at least one risk factor. **Numerator:** Out of those in the denominator, 5,496 have a referral history for testing albumin:creatinine ratio (or protein:creatinine ratio) in the urine, in the previous 12 months. **Calculation:** Numerator/denominator) * 100 **Indicator result:** 28.6%
12	CKD	NM213	The percentage of CKD‐coded patients, who are currently treated with a lipid‐lowering therapy.	Patients with CKD category G1 or G2 or unspecified.	**Testing (evaluation) date**: 31/12/2023 **Denominator:** 14,401 patients are CKD stage 3–5. **Numerator:** Out of those in the denominator, 9103 are currently treated with a lipid‐lowering therapy such as Atorvastatin, PCSK9 inhibitors and Inclisiran. **Calculation:** Numerator/denominator) * 100 **Indicator result:** 63.2%
13	CKD	NM214	The percentage of non‐CKD‐coded patients prescribed long‐term (chronic) oral nonsteroidal anti‐inflammatory drugs (NSAIDs) who have had an eGFR measurement in the preceding 12 months.	No exclusions apply to this indicator.	**Testing (evaluation) date**: 31/12/2023 **Denominator:** 1236 patients (excluding those that are CKD coded) are prescribed long‐term (chronic) oral nonsteroidal anti‐inflammatory drugs (NSAIDs). **Numerator:** Out of those in the denominator, 56 have had an eGFR measurement in the preceding 12 months. **Calculation:** Numerator/denominator) * 100 **Indicator result:** 4.5%
14	CKD	NM215	The percentage of newly CKD‐coded patients with stage G3–G5, within the preceding 12 months, who had eGFR measured on at least two occasions separated by at least 90 days, and the second test within 90 days before the diagnosis.	Patients with CKD category G1 or G2 or unspecified.	Not performed
15	CKD	NM216	The percentage of newly CKD‐coded patients with stage G3–G5, within the preceding 12 months, who had eGFR and ACR (urine albumin to creatinine ratio) measurements recorded within 90 days before or after diagnosis.	Patients with CKD category G1 or G2 or unspecified.	Not performed
16	CKD	NM247	The percentage of CKD‐coded patients with an albumin‐to‐creatinine ratio (ACR) of 500 mg/gr or more, without diabetes, who are currently treated with an ARB or an ACE inhibitor.	Patients with CKD category G1 or G2 or unspecified.	Not performed
17	CKD	NM84	The percentage of CKD‐coded patients who have hypertension and proteinuria and who are currently being treated with an ARB or an ACE inhibitor.	Patients with CKD category G1 or G2 or unspecified.	Not performed

Abbreviations: ACE, angiotensin‐converting enzyme; ACR, albumin to creatinine ratio; AF, atrial fibrillation; ARB, angiotensin receptor blocker; CG, contextualized guideline; CKD, chronic kidney disease; eGFR, estimated Glomerular filtration rate; FBC, full blood count; LFT, liver function tests; NICE, National Institute for Health and Care Excellence in the United Kingdom; NSAIDs, nonsteroidal anti‐inflammatory drugs; PCSK9, proprotein convertase subtilisin/kexin type 9; TC, thyroid cancer.

a Risk factors for indicator NM109: persistent invisible haematuria, structural renal abnormalities (such single kidney, horseshoe kidney, nephrocalcinosis, renal stones disease, dysmorphic or pelvic kidneys), functional renal abnormalities (Nephrogenic diabetes insipidus, Renal Tubular Acidosis type 1 and 2) and hereditary renal diagnoses (familial cystic disease such as autosomal dominant polycystic disease, medullary cystic disease, tuberous sclerosis, von Hippel Lindau; Familial haematurias such as C3 glomerulopathy, CFHR5 nephropathy and thin basement membrane disease/Alport spectrum disease; Fabry's disease; nephronophthisis).

### 3.6 Public consultation

**Figure 3:**
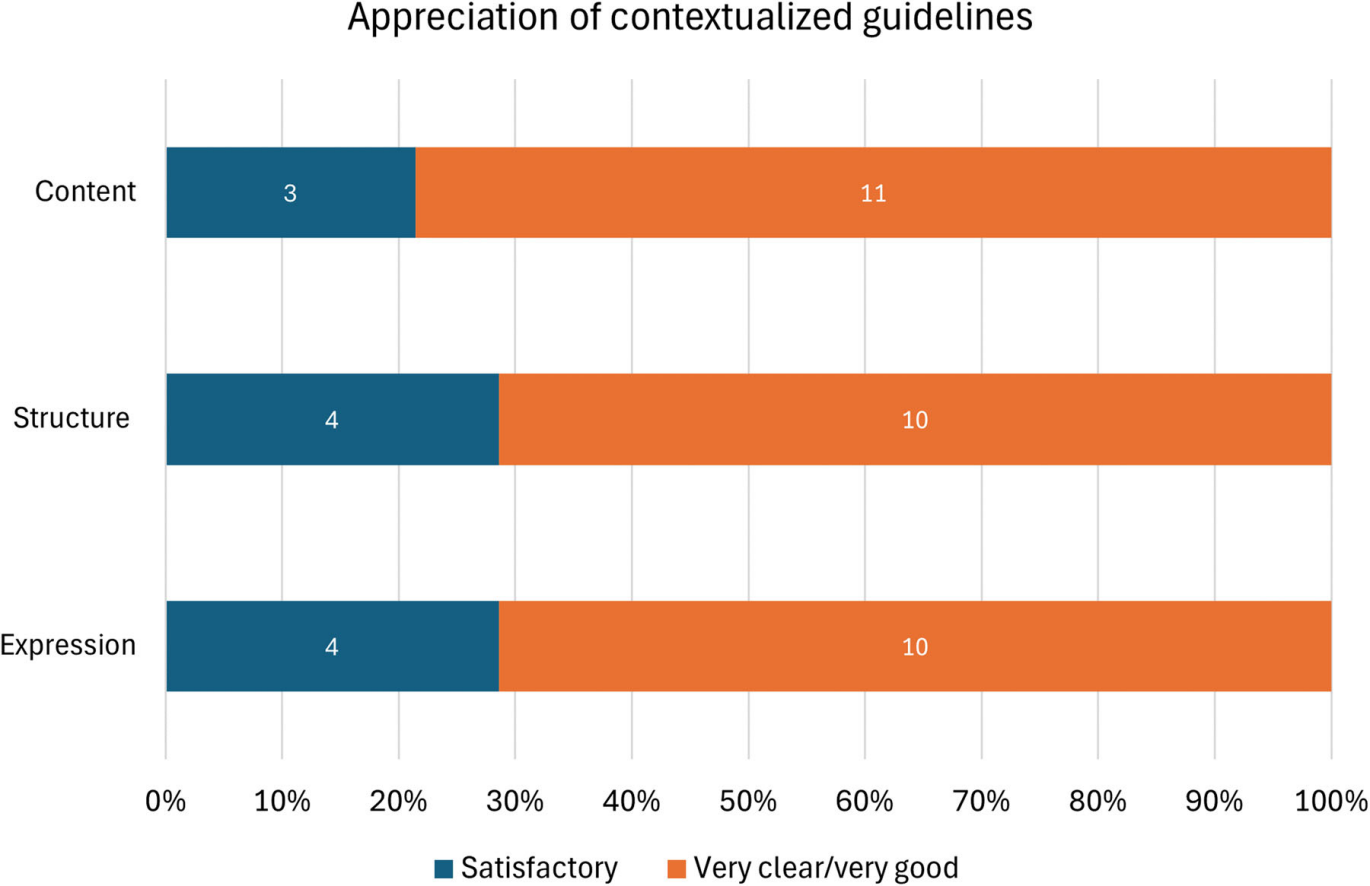
Stakeholders' overall feedback for the contextualized guidelines: Feedback of responders to contextualized guidelines during public consultation.

Public consultation was carried out according to the public and stakeholder consultation plan developed by each TEC. Invitations were sent out in month 7 of the contextualization process for two guidelines and in month 8 for the CKD guideline. The mean duration of consultation was 31 days and brief extensions (7 days) were provided to allow for additional comments. The overall participation rate was low (20%) although there was a marked improvement between the participation rate in the public consultation for the contextualization of the AF guideline (9%) and the participation rate in the public consultation for subsequent guideline contextualizations (TC: 27%, CKD: 23%). Most feedback was positive in terms of guideline content, expression and structure. Specifically, out of 14 completed feedback questionnaires, the content of the guideline was rated as (at least) ‘very clear’ by most responders (78.6%), while a similarly high percentage of responders (71.4%) rated both structure and expression of the guideline as ‘very good’ (Figure 3). No participant rated the content, structure or expression as ‘not satisfactory’. A minority of responders (35.7%) considered that the contextualized version of the guideline did not cover all the relevant areas and in several cases, specific suggestions for modifications or additions to the contextualized guidelines were made. These suggestions were reviewed by the TEC committees during the post‐consultation meetings and 36.4% of the specific suggestions (16.7% for AF, 42.9% for Thyroid and 44.4% for CKD) were considered justified and of contextual nature and were adopted. Examples of recommendations modified following public consultation are presented in Supporting Information S1: Table S3.

## 4 DISCUSSION

Following a systematic and structured process, HIO and UCY (Secretariat) organized TECs for the contextualization of three NICE guidelines and relevant quality indicators. The process ran smoothly over a period of 2 years with the involvement of medical societies, patient representatives and other scientific and healthcare stakeholders. The guidelines for AF, TC and CKD and the related quality indicators are now available for use.^20^, ^21^

This successful collaboration of Cypriot institutions with NICE demonstrates that (a) Contextualization of NICE guidelines is a timely and efficient process for countries, with limited relevant capability and capacity, to adapt existing guidelines; (b) The NICE contextualization process also offers training opportunities for those involved (expert committees, supporting scientific and administration groups and local institutions). In addition to the formal training sessions, the entire feedback provided at the different review points was a continuous learning experience. This experience enabled the Secretariat to prepare for the adaptation or development of guidelines. This is extremely important given current plans to establish a national centre for evidence‐based clinical practice in Cyprus; and (c) The contextualization of the first three guidelines, under the supervision and guidance of a world‐renowned institution such as NICE, enhances their acceptability among health professionals and stakeholders in Cyprus. Equally important has been the development of strong beliefs that such endeavours are feasible in Cyprus and worth the time and allocated resources. The feasibility and importance of guideline adaptation in the local or a resource‐limited setting has also been documented in recent literature, using different variations of the ADAPTE process or the GRADE‐ADOLOPMENT methodology.^22^, ^23^, ^24^, ^25^

Nevertheless, the implementation of a contextualization process in Cyprus was not without challenges. One important limitation of the Cyprus healthcare system is the lack of cost‐effectiveness data that could better inform a guideline contextualization process.^26^ Even so, for recommendations that were underpinned by cost‐effectiveness evaluations relevant to the NHS context, the TEC requested and received data on resource costs in Cyprus to support any modifications (e.g., modification #2 in Table 1). Future work can improve the availability of cost‐effectiveness information in Cyprus and include local data on resource use and treatment effectiveness. However, regardless of this limitation, in cases of recommendations involving specific medications, it is noted that HIO follows a flexible drug reimbursement approach that allows, in cases of more expensive (and more effective) medication, to offer the medication with a co‐payment by the patient. As such, from a healthcare system perspective, the cost‐effectiveness of these recommendations can be maintained. Other limitations included challenges regarding meeting attendance by TEC members and stakeholder participation in the consultation phase. Specifically, while efforts were made to enhance TEC meeting attendance through favourable scheduling, a small reimbursement package, frequent reminders and same‐day notifications, meeting attendance was high but not optimal. The HIO and Secretariat aim to improve attendance through personal communications with nonattending members and the option to invite replacement members. In addition, stakeholder participation in the consultation phase was generally low. Although this increased considerably between the first and second year, it remained low (<30% participation rate). This can be attributed to the unfamiliarity of the stakeholders with the contextualization process, as well as to the overall lack of guideline and quality indicators implementation in the healthcare system of Cyprus. The implementation of the contextualized guidelines and quality indicators in the clinical setting of Cyprus and the adaptation of more guidelines are expected to further improve this metric in the coming years. Nonetheless, additional strategies to enhance engagement of stakeholders such as early involvement, availability of user‐friendly and alternative communication channels as well as provision of educational and training resources should be considered in the future.^27^, ^28^, ^29^ These may be especially helpful with stakeholders unfamiliar with guideline development. In addition, the use of open and targeted invitations to take part in the consultation process has the potential to increase the involvement of healthcare professionals. Increased involvement will further enhance the transparency of the guideline contextualization and provide feedback on how the stakeholder input influences the final recommendations.^30^

The successful contextualization of three NICE guidelines paves the way for the adaptation of other NICE guidelines in priority fields identified from existing data in the Cyprus healthcare system. In 2025, it is expected that HIO will adopt the NICE guidelines NG192 on Caesarean delivery and NG136 on Hypertension in adults: diagnosis and management. In Cyprus, caesarean section deliveries occur in well above 50% of births on the island^31^ while hypertension affects 26% of women and 35.7% of men with approximately half of the affected individuals receiving appropriate treatment.^32^

To tackle the most common barriers in guideline implementation in the clinical setting,^33^ additional steps in the form of educational, administrative and technological interventions are scheduled for the upcoming years. More importantly, HIO will monitor the changes in clinical practice and outcomes on an annual basis using the developed quality indicators, as described in other settings.^34^ Lastly, the experience gained through the collaboration with HIO in Cyprus, allows NICE to better inform its tools and services towards sharing best practices and expertise in other resource‐limited settings.

## 5 CONCLUSION

Since 2022, three NICE guidelines and 17 quality indicators have been successfully contextualized for the Cyprus healthcare system by HIO through a systematic process that involved key local healthcare and societal stakeholders. The contextualization process was quality‐assured by NICE through a supervised adaptation process. This positive experience demonstrates the feasibility of this approach in small countries, while it also highlights the benefits of capacity and capability building in countries with limited experience in developing, adapting and implementing evidence‐based guidelines.

## CONTEXTUALIZATION GROUP

George Athanasiou, Elias Avraam, Mary Avraamidou, Anastasios Bagourdis, Charis Charilaou, Stelios Christodoulou, Natasa Christofides, Theodoros Christophides, Vasilis Constantinidis, Dimitris Dimitriou, Stella Eleftheriadou, Christina Flourou, Savvas Frangos, Kyriakos Frantzeskou, Costas Hoplaros, Kyriakos Ioannou, Nikolaos Kadoglou, Chara Kalogirou, Vasileios Karathanos, Andreas Kostis, Andreas Kousios, Ekaterini Lambrinou, Emily Mavrokordatou, Michael Michael, Nicos Mitsides, Chrystalleni Mylonas, Georgia Orphanou, Elias Papasavvas, Doros Polydorou, Stavros Stavrou, Elena Theofanous, Despo Theodorou, Giannis Thrasyvoulou, Chryssa Tziakouri‐Shakalli, Theodora Vassiliou, Alexis Vrachimis.

## AUTHOR CONTRIBUTIONS

**Panayiotis Kouis**: Investigation; project administration; methodology; data curation; visualization; formal analysis; writing—original draft preparation. **Hugh McGuire**: Conceptualization; methodology; formal analysis; supervision; writing—original draft preparation. **Monika Kyriacou**: Conceptualization; methodology; data curation; project administration; writing—review and editing. **Anneza Yiallourou**: investigation; project administration; data curation; writing—review and editing. **Anastasis Sioftanos**: Investigation; data curation; writing—review and editing. **Ourania Kolokotroni**: Investigation; data curation; writing—review and editing. **Haris Achilleos**: Investigation; data curation; writing—review and editing. **Craig Grime**: methodology; writing—review and editing. **Pilar Pinilla‐Domínguez**: Methodology; project administration; writing—review and editing. **Giorgos Giallouros**: Investigation; data curation; project administration; writing—review and editing. **Christina Englezou**: Investigation; data curation; project administration; writing—review and editing. **Contextualization Group**: Investigation; writing—review and editing. **Panayiotis K. Yiallouros**: Conceptualization; methodology; writing—review and editing. **Georgios K. Nikolopoulos**: Conceptualization; project administration; methodology; formal analysis; writing—original draft preparation; supervision.

## CONFLICT OF INTEREST STATEMENT

The authors declare no conflict of interest.

## ETHICS STATEMENT

This article describes the methodology and experience for contextualizing clinical guidelines and did not involve research with human participants; hence ethics approval was not applicable.

## Supporting information

Supporting information.

Supporting information.

## Data Availability

The data that support the findings of this study are available from the corresponding author upon reasonable request.
