# Supplementary material for: Contextualizing guidelines for the health system of Cyprus: Experiences and lessons learnt
Source: Clin Public Health Guidel. 2025 Feb 5;2(2):e70013. doi: 10.1002/gin2.70013 (PMC13563364; doi:10.1002/gin2.70013)
Supplement: Supplementary file 1 [file GIN2-2-e70013-s001.docx]

**Supplementary Table 1:** Elements of contextualization procedure overseen by NICE.

| **Contextualization procedure element** | **NICE role** |
| --- | --- |
| Proposed membership in the TEC | The proposed membership is prepared by HIO in Cyprus. NICE ensures that consideration has been given to the specialist roles and expertise needed to make decisions. |
| Draft guideline scope | The draft guideline scope prepared by the TEC in Cyprus. NICE considers the scope for consistency with the evidence reviews underpinning the NICE guideline to determine if they cover the new scope adequately. |
| Draft changes to the recommendations and associated justification | The draft changes to the recommendations and associated justification prepared by the TEC in Cyprus. NICE checks for consistency with the agreed scope, and that the contextualized or new recommendations are consistent with existing evidence reviews. NICE also considers if the rationale or justification for the contextualized or new recommendations are adequate. |
| Final guideline following the public consultation period | The final guideline prepared by the TEC following the public consultation period. NICE reviews the changes made to the draft recommendations based on the consultation responses to ensure it is in line with existing evidence and that adequate justification has been provided. |
| Derived quality indicators and validity assessments | NICE provides quality assurance on the proposed purpose of any indicators and the outcomes that are intended to be improved. In addition, NICE oversees the methodology being used to develop and test the quality indicators. |

TEC: Technical Expert Committee, HIO: Health Insurance Organization

**Supplementary Table 2:** Excluded NICE indicators from the contextualization process for AF, TC and CKD guidelines

| **#** | **CG** | **NICE reference** | **NICE indicator title** | **Reason for exclusion** |
| --- | --- | --- | --- | --- |
| 1 | AF | CCG56 | Proportion of patients admitted to hospital for stroke with a pre-existing diagnosis of atrial fibrillation, who were not on anticoagulation | **Overlapping** with selected NICE indicator CCG55 “Proportion of patients admitted to hospital for stroke with a pre-existing diagnosis of atrial fibrillation, who were on anticoagulation” |
| 2 | AF | NM164GP | The contractor establishes and maintains a register of patients with atrial fibrillation, including patients with ‘AF resolved’ | **Technical and institutional limitations** as individual practices are not set-up to maintain databases/registries for specific diseases. |
| 3 | AF | NM180GP | The percentage of patients with one or more of the following conditions: CHD, atrial fibrillation, chronic heart failure, stroke or TIA, diabetes or dementia who have been screened for hazardous drinking using the FAST or AUDIT-C tool in the preceding 2 years. | **Limited utility and technical difficulties** of this indicator as the prevalence of alcoholism in Cyprus is low and the HIO electronic data collection system does not include the FAST or AUDIT-C tools for data collection. |
| 4 | AF | NM181GP | The percentage of patients with one or more of the following conditions: CHD, atrial fibrillation, chronic heart failure, stroke or TIA, diabetes or dementia with a FAST score of 3 or more or AUDIT-C score of 5 or more in the preceding 2 years who have received brief intervention to help them reduce their alcohol related risk within 3 months of the score being recorded | **Limited utility and technical difficulties** of this indicator as the prevalence of alcoholism in Cyprus is low and the HIO electronic data collection system does not include the FAST or AUDIT-C tools for data collection. |
| 5 | TC | CCG02 | The proportion of new cases of cancer with stage at diagnosis recorded. | **Technical difficulties** for including cancer stage as part of the HIO electronic data collection system. |
| 6 | TC | CCG03 | The proportion of cancer cases detected at stage 1 or 2. | **Technical difficulties** for including cancer stage as part of the HIO electronic data collection system. |
| 7 | TC | NM 204 | The percentage of patients with cancer, diagnosed within the preceding 12 months, who have had a discussion within 3 months of diagnosis about the support available from primary care. | **Institutional limitation** as there is no structured holistic support system available for cancer patients in Cyprus. |
| 8 | TC | NM205 | The percentage of patients with cancer, diagnosed within the preceding 24 months, who have a patient Cancer Care Review using a structured template within 12 months of diagnosis. | **Institutional limitation** as the is no Cancer Care Review template in Cyprus. |
| .9 | TC | NM234 | The contractor establishes and maintains a register of all cancer patients excluding non-melanotic skin cancers. | **Technical and institutional limitations** as individual practices are not set-up to maintain databases/registries for specific diseases |
| 10 | CKD | NM83 | The contractor establishes and maintains a register of patients aged 18 years or over with CKD with classification of categories G3a to G5 (previously stage 3 to 5). | **Technical and institutional limitations** as individual practices are not set-up to maintain databases/registries for specific diseases |
| 11 | CKD | NM217 | The percentage of patients on the CKD register and with an albumin to creatinine ratio (ACR) of less than 70 mg/mmol, without moderate or severe frailty, in whom the last blood pressure reading (measured in the preceding 12 months) is less than 135/85 mmHg if using ambulatory or home monitoring, or less than 140/90 mmHg if monitored in clinic. | **Technical difficulties** to quantify frailty in the HIO electronic data collection system. |
| 12 | CKD | NM246 | The percentage of patients on the CKD register and currently treated with an ARB or an ACE inhibitor who are also currently treated with an SGLT2 inhibitor if they have either: no type 2 diabetes and a urine ACR of 22.6 mg/mmol or more, or type 2 diabetes and a urine ACR 3 mg/mmol or more. | **Technical difficulties** to implement this indicator at the HIO electronic data collection system as the test results relevant to this indicator are currently available in PDF format and not in a fully digital (e.g.XML) format to allow for easy automatic data collection. |
| 13 | CKD | NM248 | The percentage of patients on the CKD register and with an albumin to creatinine ratio (ACR) of 70 mg/mmol or more, without moderate or severe frailty, in whom the last blood pressure reading (measured in the preceding 12 months) is less than 125/75 mmHg if using ambulatory or home monitoring, or less than 130/80 mmHg if monitored in clinic. | **Technical difficulties** to quantify frailty in the HIO electronic system electronic data collection system. |

CG: Contextualized guideline; AF: Atrial Fibrillation; TC: Thyroid Cancer; CKD: Chronic Kidney Disease; CHD: Coronary Heart Disease; ACR: albumin to creatinine ratio; ARB: Angiotensin receptor blocker; SGLT2: Sodium-glucose co-transporter-2 inhibitors; PDF: Portable Document Format; XML: Extensible Markup Language format

**Supplementary Table 3:** Examples of recommendations modified or not after public consultation

| **#** | **CG** | **Recommendation**  **(# CG)** | **Pre-consultation wording** | **Public consultation comment** | **TEC decision and justification** | **Post-consultation wording** |
| --- | --- | --- | --- | --- | --- | --- |
| 1 | AF | 1.2.2  (1.2.2) | Assess the risk of bleeding when:   - considering starting anticoagulation in people with atrial fibrillation **and** - reviewing people already taking anticoagulation.   Use the [ORBIT bleeding risk score](https://www.mdcalc.com/orbit-bleeding-risk-score-atrial-fibrillation) because evidence shows that it has a higher accuracy in predicting absolute bleeding risk than other bleeding risk tools. Accurate knowledge of bleeding risk supports shared decision making and has practical benefits, for example, increasing patient confidence and willingness to accept treatment when risk is low and prompting discussion of risk reduction when risk is high. Although ORBIT is the best tool for this purpose, other bleeding risk tools, such as the HAS-BLED score, may need to be used until it is embedded in clinical pathways and electronic systems. | “contextualized guideline should enable treating physicians to use the HAS-BLED score” | From the initial contextualization stage, TEC was aware that current practices to evaluate risk of bleeding in atrial fibrillation rely on the use of HAS-BLED score. However, based on the available evidence, it was decided to suggest the use of ORBIT as the main tool to evaluate risk of bleeding as it was found to be more sensitive compared to HAS-BLED. To this effect, the TEC considered that the use of ORBIT as more sensitive tool for the evaluation of the absolute risk of bleeding is a real advantage for supporting patients and clinicians in joint decision-making and advocated in favor of including it the contextualized guideline. However, given that ORBIT is not part of the current clinical pathways in Cyprus and is not part of the HIO electronic system, the guideline allows for the use of **other tools, such as the HAS-BLED score,** until ORBIT is fully incorporated in clinical practice and the electronic systems. In response to this comment, the TEC agreed to make the corresponding change in point 1.2.2 of the contextualized guideline. | Assess the risk of bleeding when:   - considering starting anticoagulation in people with atrial fibrillation **and** - reviewing people already taking anticoagulation.   Use the [ORBIT bleeding risk score](https://www.mdcalc.com/orbit-bleeding-risk-score-atrial-fibrillation) because evidence shows that it has a higher accuracy in predicting absolute bleeding risk than other bleeding risk tools. Accurate knowledge of bleeding risk supports shared decision making and has practical benefits, for example, increasing patient confidence and willingness to accept treatment when risk is low and prompting discussion of risk reduction when risk is high. Although ORBIT is the best tool for this purpose, other bleeding risk tools, such as the HAS-BLED score, may need to be used until it is embedded in clinical pathways and electronic systems. |
| 2 | AF | Not applicable | Not applicable | “Guideline being more focused to treatment compared to diagnosis” | The TEC acknowledges that the scope of the guideline is primarily focused on treatment of atrial fibrillation instead of diagnosis. However, this is the result of the current situation in the clinical setting. Diagnosis of atrial fibrillation is straightforward, easy and is not characterized by any significant uncertainties or variation in practice. On the contrary, management of AF is more challenging, variations in practice exist and additional factors like patient safety and comorbidities must be taken into account. This is reflected in the guideline scope and text that is primarily focused on treatment compared to diagnosis. In addition, the TEC emphasized that the comment itself was quite general, and it was unclear if there was a specific diagnostic aspect or procedure that was considered to be not adequately covered. In relation to this comment the TEC was informed of the instructions that were given to the participants of the consultation process, to provide specific comments, supported by relevant evidence. In summary, in response to this comment, the TEC decided to not make any further edits to the guideline | Not applicable |
| 3 | TC | Rationale and impact section  Recommendations 1.5.1-1.5.6  (Recommendations 1.5.1-1.5.6) | The committee agreed that if thyroglobulin antibodies are not detected, then thyroglobulin levels can be interpreted at face value. In such a case, this initial evidence of recurrence from thyroglobulin testing should lead to further investigations, either to confirm or refute recurrence. They also recommended that people who have previously been cleared of having recurrence after a thyroglobulin test, but now have rising thyroglobulin levels, should also have further investigations for recurrence. This is because the rise in thyroglobulin levels might be a ‘new’ sign of recurrence that requires investigation. Further investigations could include neck ultrasound, CT scan of neck and chest or MRI scan of the neck. The choice often depends on local availability, and a prior knowledge of what happened with the patient in assessing where in the body recurrence is most likely to be. | “In the rationale text 1.5.1 -1.5.6, the committee did not include FDG-PET CT scan especially for well-differentiated cancers that are non-iodine-avid (i.e. Hurthle-cell carcinoma) or cancer that have no clinical indication of de-differentiation” | The TEC reviewed this comment about additional testing using FDG-PET CT scan, especially for well-differentiated cancers that are non-iodine-avid (i.e. Hurthle-cell carcinoma) or cancers that have no clinical indication of de-differentiation. In response to this comment, the TEC acknowledges that there is some utility of FDG-PET CT scan in these cases although evidence in scarce in order to be included in the main body of the guideline. In this respect, the TEC decided to edit the following sentence in the rationale section: | The committee agreed that if thyroglobulin antibodies are not detected, then thyroglobulin levels can be interpreted at face value. In such a case, this initial evidence of recurrence from thyroglobulin testing should lead to further investigations, either to confirm or exclude recurrence. They also recommended that people who have previously been cleared of having recurrence after a thyroglobulin test, but now have rising thyroglobulin levels, should also have further investigations for recurrence. This is because the rise in thyroglobulin levels might be a ‘new’ sign of recurrence that requires investigation. Further investigations could include neck ultrasound, CT scan of neck and chest or MRI scan of the neck and iodine 131 scintigraphy with stimulated TSH. In case of non-iodine-avid disease, FDG PET/CT can be considered. The choice often depends on local availability, and a prior knowledge of what happened with the patient in assessing where in the body recurrence is most likely to be. |
| 4 | TC | Not applicable | Not applicable | “Include using dosimetry and its protocols in metastatic cancers (lung, brain) and highlight usefulness of special probes for dosimetry” | The TEC highlights that dosimetry is already presented in Table under point 1.3.20 and the additional points raised “addition of points regarding preparations of nuclear medicine practitioners” are considered out of scope. In addition, the inclusion of details regarding nuclear medicine protocols is out of the scope of this guideline which is targeting all specialties involved in the care of thyroid cancer patients. | Not applicable |
| 5 | CKD | 1.6.9  (1.6.11) | For adults with CKD but without diabetes:   - refer for nephrology assessment and offer an ARB or an ACE inhibitor (titrated to the highest licensed dose that they can tolerate), if ACR is 500 mg/g or more. - Consider SGLT-2 inhibitors for treating chronic kidney disease (CKD) in adults. It is recommended only if:   - it is an add-on to optimised standard care including the highest tolerated licensed dose of angiotensin-converting enzyme (ACE) inhibitors or angiotensin-receptor blockers (ARBs), unless these are contraindicated, and   - people have an estimated glomerular filtration rate (eGFR) of 25 ml/min/1.73 m^2^ to 75 ml/min/1.73 m^2^ at the start of treatment and:   - have a urine albumin-to-creatinine ratio (ACR) of 30 mg/gr or more. | “For point 1.6.11 and the recommendation to [Consider SGLT-2 inhibitors…], most recent KDIGO guidelines of 2024 recommend SGLT2 inhibitors as first-line treatment” | The TEC agrees with this comment. Based on the most recent summary of evidence to its disposal (presented as summary forest plot in Figure 22 in page S215 (bottom section-no diabetes) of the most recent KDIGO guidelines and especially from well conducted studies such as the DAPA−CKD and EMPA-KIDNEY, SGLT2 inhibitors are associated with important benefits for the patients. As a result the wording in recommendation 1.6.11 has been changed accordingly.  In addition to be consistent with recommendation 3.7.2 of the most recent KDIGO guidelines, the eGFR and ACR limits have been changed from “25 ml/min/1.73 m^2^ to 75 ml/min/1.73” m^2^ and “30 mg/gr” to “20 ml/min/1.73 m^2^“ and “200 mg/gr” respectively. | For adults with CKD but without diabetes:   - refer for nephrology assessment and offer an ARB or an ACE inhibitor (titrated to the highest licensed dose that they can tolerate), if ACR is 500 mg/g or more. - Offer SGLT-2 inhibitors for treating chronic kidney disease (CKD) in adults. It is recommended only if:   - it is an add-on to optimised standard care including the highest tolerated licensed dose of angiotensin-converting enzyme (ACE) inhibitors or angiotensin-receptor blockers (ARBs), unless these are contraindicated, and   - people have an estimated glomerular filtration rate (eGFR) of 20 ml/min/1.73 m^2^ ~~to 75 ml/min/1.73 m~~^~~2~~^ at the start of treatment and:   - have a urine albumin-to-creatinine ratio (ACR) of 200 mg/gr or more. |
| 6 | CKD | -  (1.6.9) | Consider Non-steroidal Mineralocorticoid Receptor Antagonists (Finerenone) for treating stage 3 and 4 chronic kidney disease (with albuminuria) associated with type 2 diabetes in adults. It is recommended only if:   - it is an add-on to optimised standard care; this should include, unless they are unsuitable, the highest tolerated licensed doses of:   - angiotensin-converting enzyme (ACE) inhibitors or angiotensin-receptor blockers (ARBs) and   - sodium–glucose cotransporter‑2 (SGLT2) inhibitors and - the person has an estimated glomerular filtration rate (eGFR) of 25 ml/min/1.73 m^2^ or more. | The range of patients that finerone can be administered to should expand to also include CKD stages 1 and 2 along with albuminuria, based on the findings of FIGARO and FIDELIO trials | The TEC assessed this point and notes that [the NICE assessment of March 2023](https://www.nice.org.uk/guidance/ta877/chapter/3-Committee-discussion) indicates that the marketing indication of fineranone is for CKD 3 and 4 with albuminuria and that's why it is used for this CKD bracket. Furthermore, the TEC noted that although findings from the two studies (Figaro and Fidelio) are indicative for positive effect also in CKD stages in 1 and 2, it also highlights that Figaro DKD showed benefit in stages 1 and 2 but only included people with moderate to severe albuminuria of an ACR od 300-5000 mg/g while Fidelio DKD also included people of CKD 1 and 2 again with ACR 300-5000 but a large fraction of the patients (55%) had an eGFR less than 45ml/min. Finally, according to the European Medical Agency (EMA) therapeutic indication for this product, finerenone is indicated for the treatment of chronic kidney disease (stage 3 and 4 with albuminuria) associated with type 2 diabetes in adults. As a result, the TEC does not agree to increase the range of patients that finerenone can be administered to and the relevant point remains as is. | Not applicable |

CG: Contextualized guideline; AF: Atrial Fibrillation; TC: Thyroid Cancer; CKD: Chronic Kidney Disease; FDG PET-CT: fludeoxyglucose-18 positron emission tomography scam; SGLT2: Sodium-glucose co-transporter-2 inhibitors; KIDGO: Kidney Disease: Improving Global Outcomes; eGFR: estimated Glomerular Filtration Rate; ACR: albumin to creatinine ratio; EMA: European Medical Agency; DKD: Diabetic Kidney Disease
